# Supplementary material for: Species and condition shape the mutational spectrum in experimentally evolved biofilms
Source: mSystems. 2023 Sep 28;8(5):e00548-23. doi: 10.1128/msystems.00548-23 (PMC10654089; doi:10.1128/msystems.00548-23)
Supplement: Fig. S1 — Biofilm productivity of four experiments. [file msystems.00548-23-s0003.pdf]

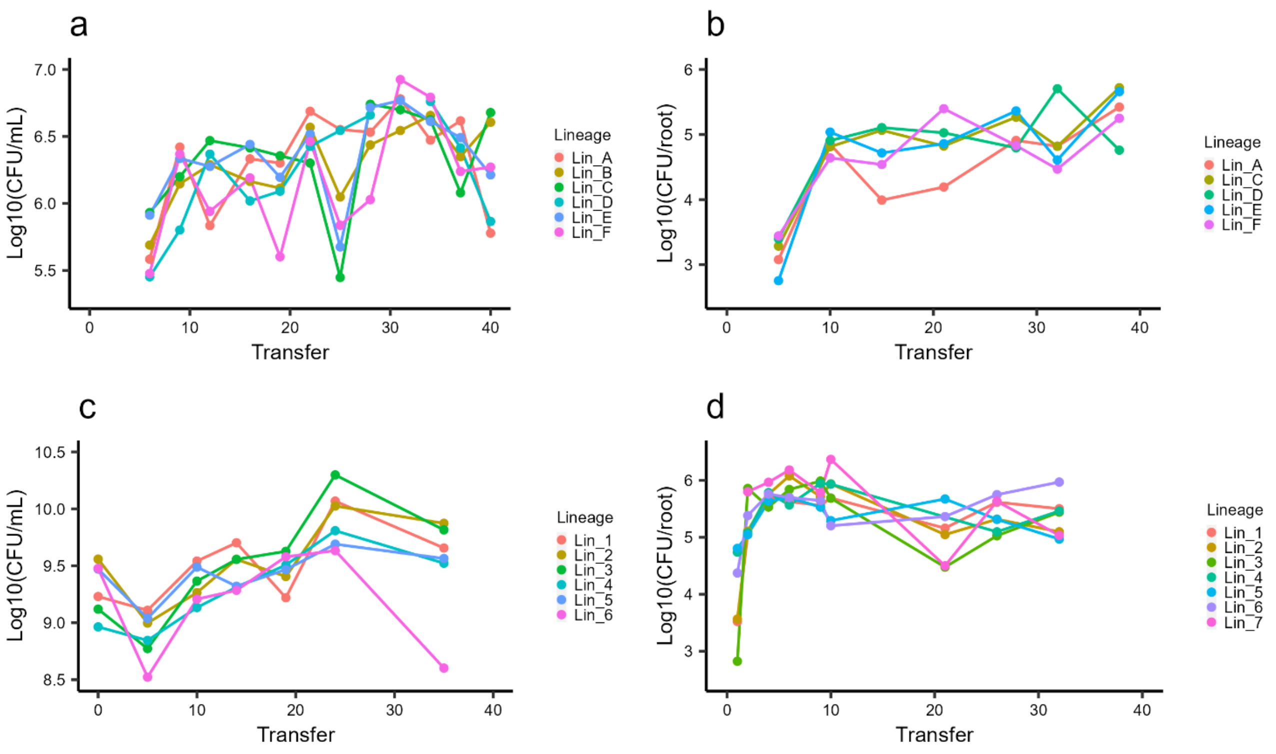

Fig S1 Biofilm productivity of four experiments. Data are from previous studies and replotted for better comparison.
