# Supplementary material for: Species and condition shape the mutational spectrum in experimentally evolved biofilms
Source: mSystems. 2023 Sep 28;8(5):e00548-23. doi: 10.1128/msystems.00548-23 (PMC10654089; doi:10.1128/msystems.00548-23)
Supplement: Fig. S3 — Estimated population size of each population and timepoints calculated from biofilm productivity data. [file msystems.00548-23-s0005.pdf]

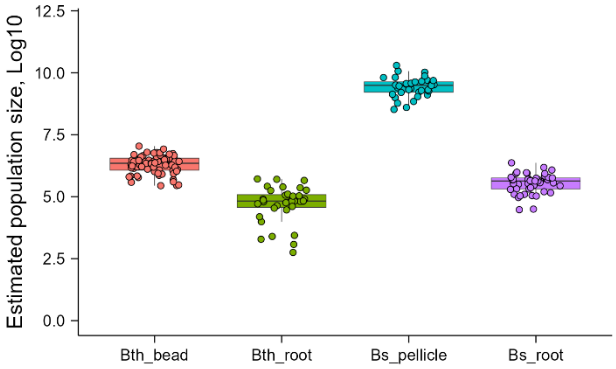

Fig S3 Estimated population size of each population and timepoints calculated from biofilm productivity data. Boxes indicate Q1–Q3, lines indicate the median, black circles filled with different color indicate the population size of each population.
