# Supplementary material for: Species and condition shape the mutational spectrum in experimentally evolved biofilms
Source: mSystems. 2023 Sep 28;8(5):e00548-23. doi: 10.1128/msystems.00548-23 (PMC10654089; doi:10.1128/msystems.00548-23)

Supplementary Fig. 5A, Bth_bead lineage diagram

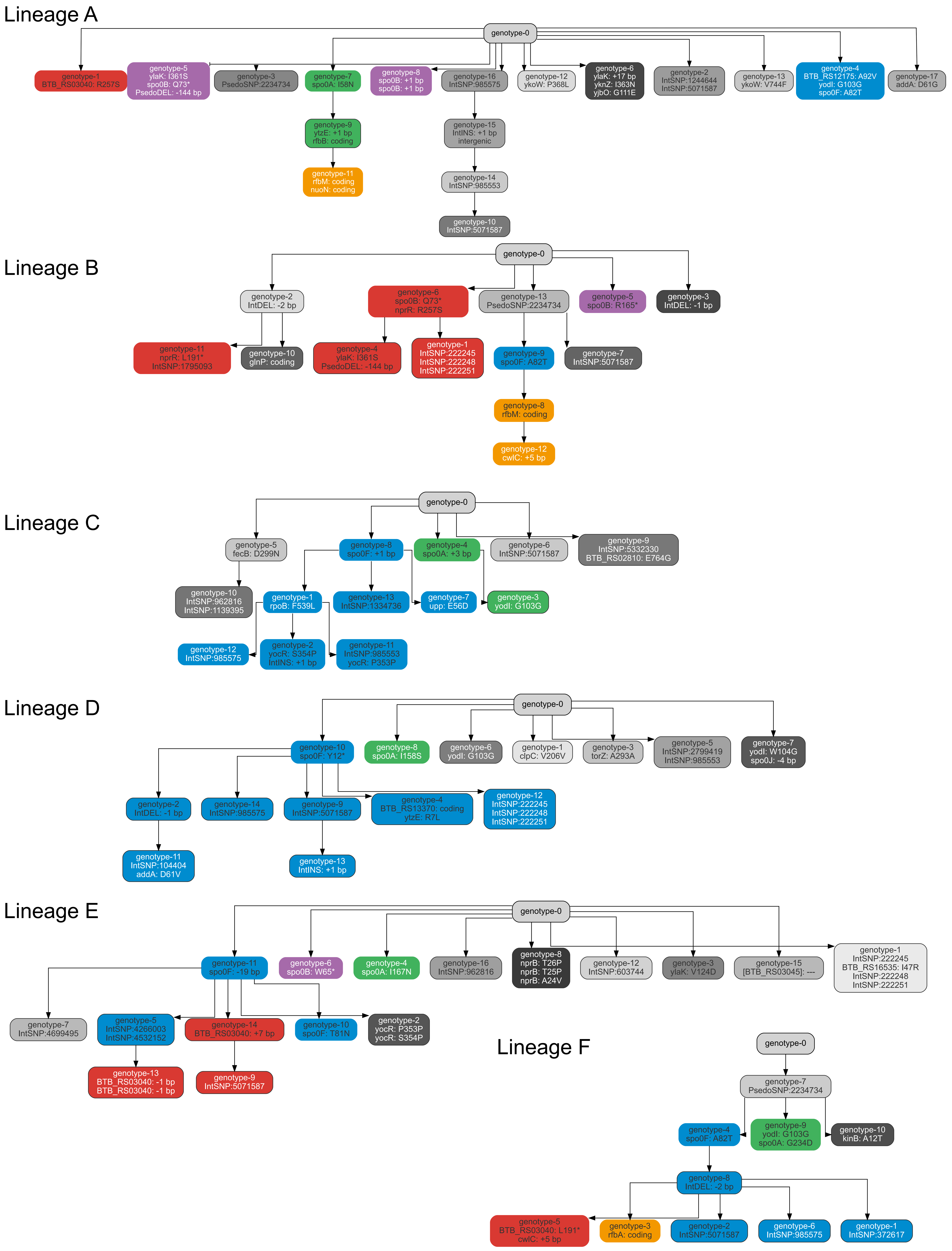

Supplementary Fig. 5B, Bth_root lineage diagram

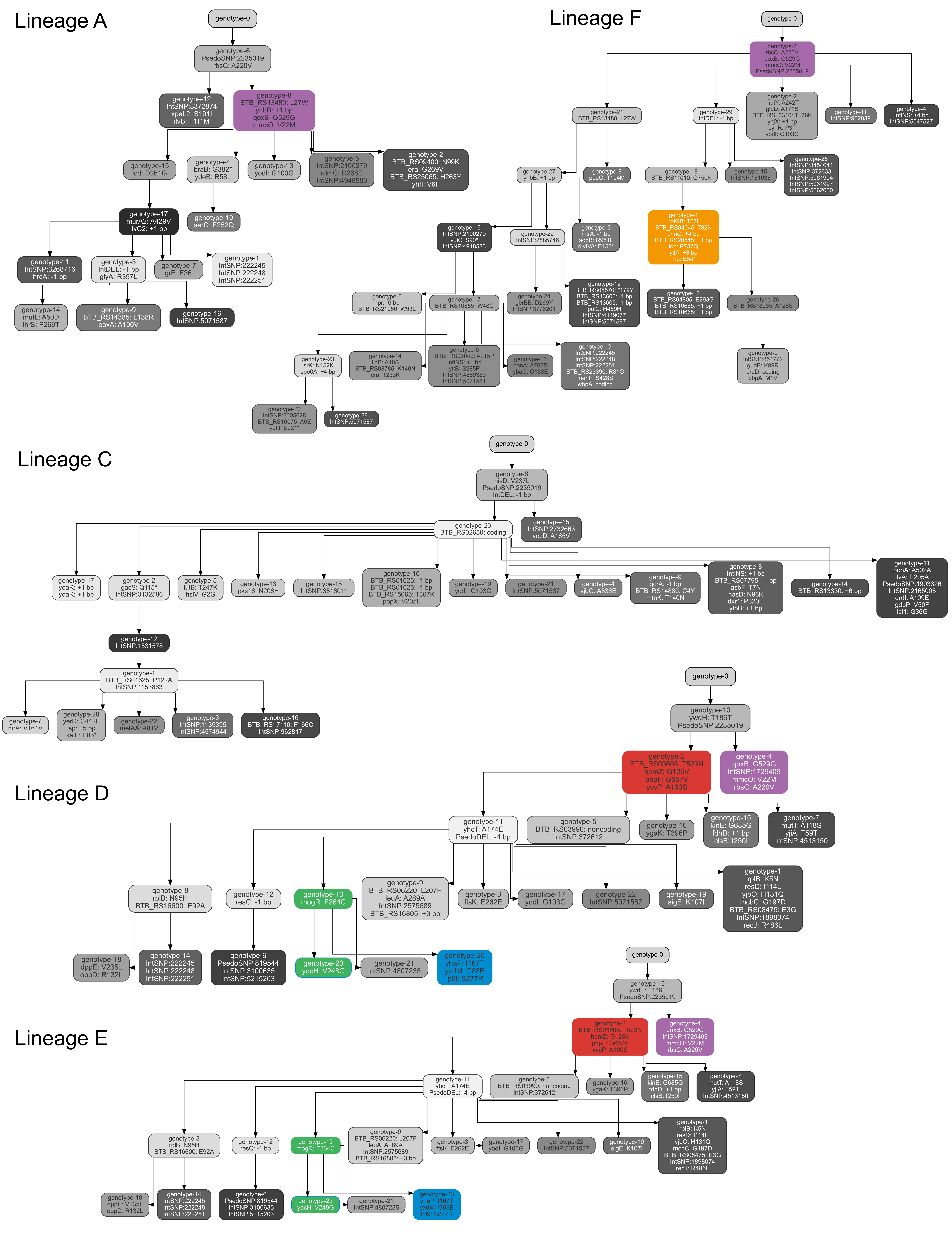

Supplementary Fig. 5C, Bs_pellicle lineage diagram

Lineage 1

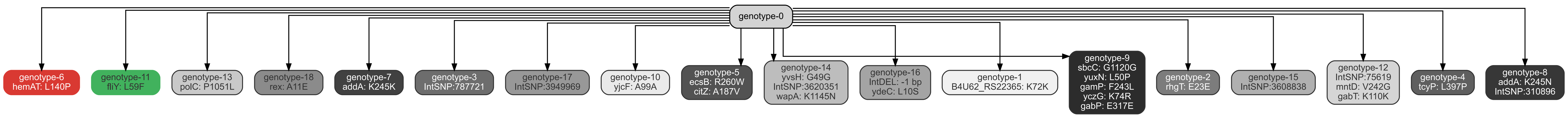

Lineage 2

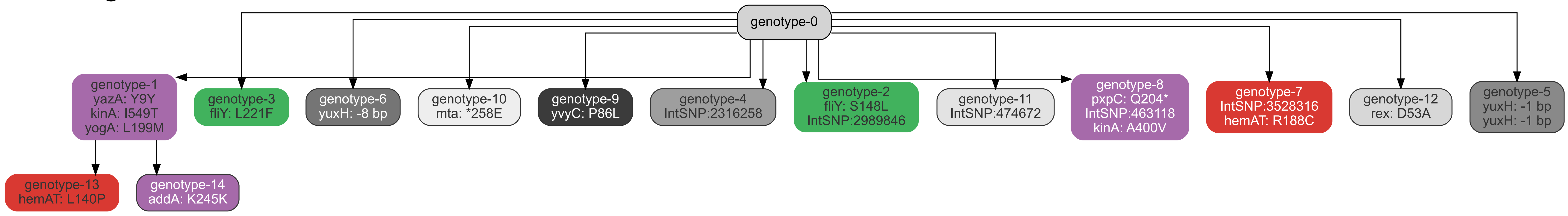

Lineage 3

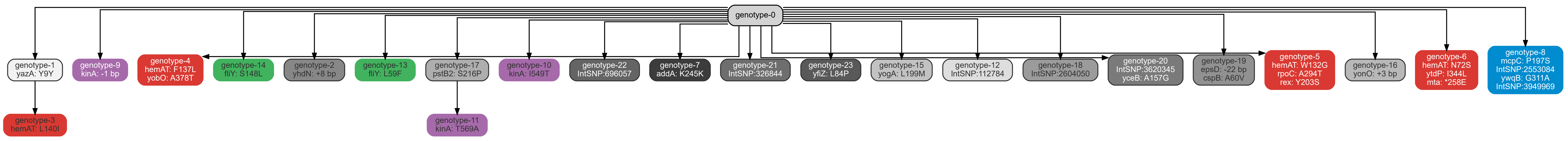

Lineage 4

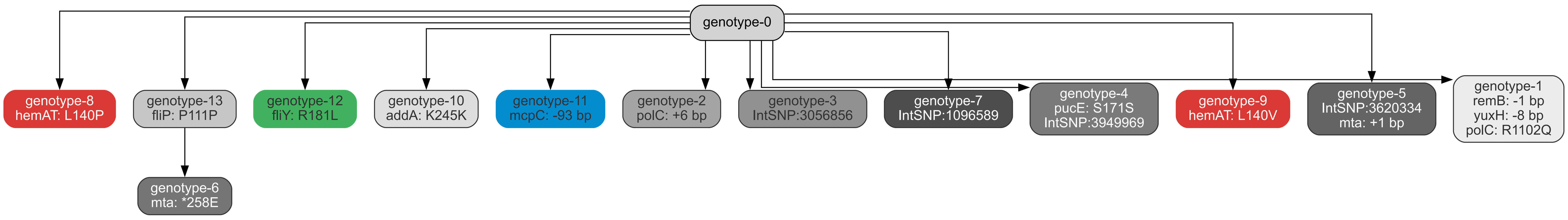

Lineage 5

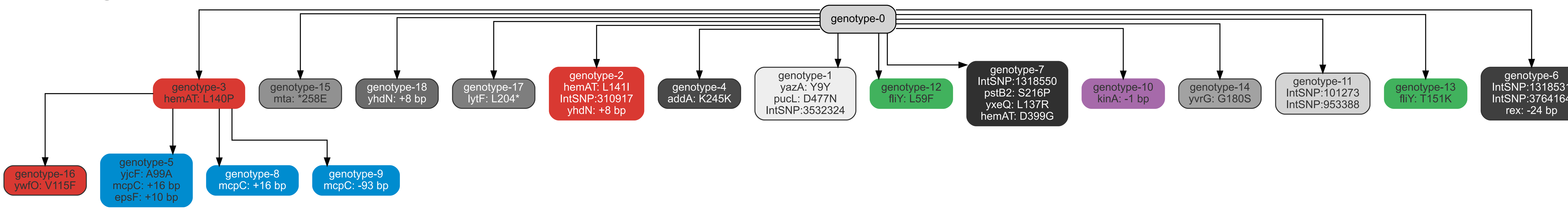

Lineage 6

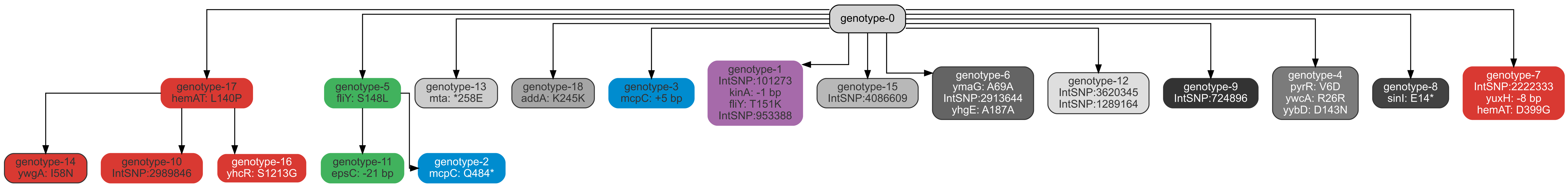

Supplementary Fig. 5D, Bs_root lineage diagram

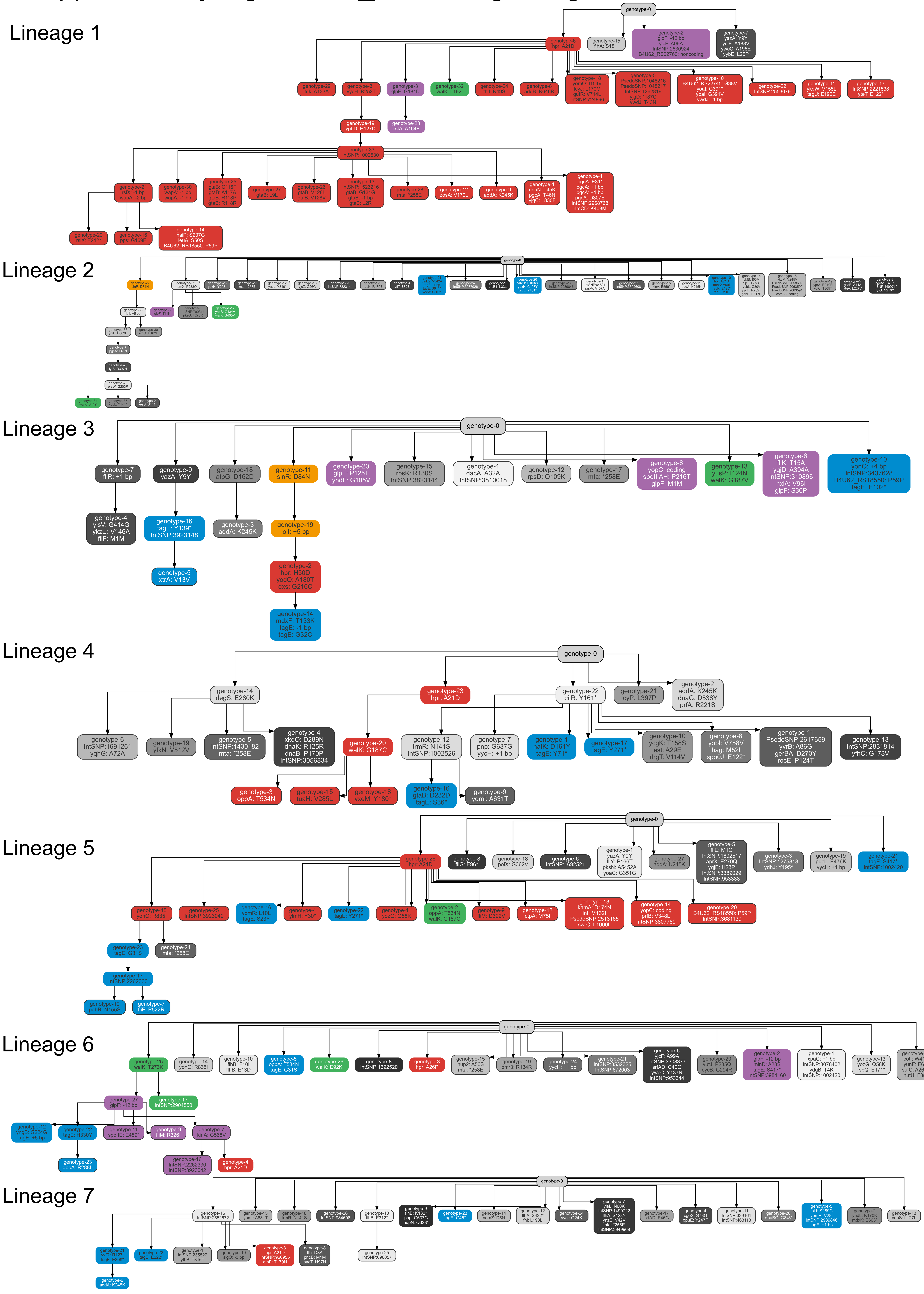

Supplement: Fig. S5 — Linage diagrams. [file msystems.00548-23-s0007.pdf]
