# Supplementary material for: Species and condition shape the mutational spectrum in experimentally evolved biofilms
Source: mSystems. 2023 Sep 28;8(5):e00548-23. doi: 10.1128/msystems.00548-23 (PMC10654089; doi:10.1128/msystems.00548-23)
Supplement: Table S1 — Laboratory evolution setups of four experiments. [file msystems.00548-23-s0008.pdf]

Table S1 Laboratory evolution setups of four experiments

**Group**	**Bth_bead**	**Bth_root**	**Bs_pellicle**	**Bs_root**
Species	*B. thuringiensis*	*B. thuringiensis*	*B. subtilis*	*B. subtilis*
Adaptation condition	Nylon beads floating in the medium	*A. thaliana* root	Pellicle biofilm at the air-medium interface	*A. thaliana* root
Medium	EPS medium	MSNg medium	MSgg medium	MSNg medium
Medium volume	1000 μL	300 μL	2000 μL	300 μL
Temperature	30 °C	16 h light at 24 °C/8 h dark at 20 °C	30 °C	16 h light at 24 °C/8 h dark at 20 °C
Transfer interval time	24h	48h	48h	48h
Number of transfers	40	38	35	32
Number of parallel linages	5	6	5	7
Timepoints	7	6	7	5
Total population samples sequenced	34	35	35	34
Adaptation model	one colonized bead to **two** new beads	one colonized root to one new root	static floating biofilm disrupted by glass beads and vortexing for 1:100 reinoculation	one colonized root to one new root
Shaking condition	90 rpm	90 rpm	static	90 rpm
