# Supplementary material for: Species and condition shape the mutational spectrum in experimentally evolved biofilms
Source: mSystems. 2023 Sep 28;8(5):e00548-23. doi: 10.1128/msystems.00548-23 (PMC10654089; doi:10.1128/msystems.00548-23)
Supplement: Table S2 — dN/dS ratio. [file msystems.00548-23-s0009.pdf]

Table S2 *dN/dS* ratio

Replicate	Bth_bead	Bth_root	Bs_pellicle	Bs_root
R1	2.98	2.85	0.50	0.79
R2	*	1.14	1.57	0.91
R3	0.68	0.88	2.85	0.57
R4	0.45	0.75	0.47	0.82
R5	2.44	2.37	1.14	1.06
R6	1.08		0.78	1.99
R7				0.93
Total ratio	1.36	1.25	0.95	0.91
Average	1.53	1.60	1.22	1.01
SD	1.00	0.85	0.82	0.43

* no synonymous mutation detected in this lineage
