# Supplementary material for: Species and condition shape the mutational spectrum in experimentally evolved biofilms
Source: mSystems. 2023 Sep 28;8(5):e00548-23. doi: 10.1128/msystems.00548-23 (PMC10654089; doi:10.1128/msystems.00548-23)
Supplement: Table S3 — Transposase gene in the B. thuringiensis 407 genome and Insertion sequence definition in the second-round analysis using breseq. [file msystems.00548-23-s0010.pdf]

Table S3

A/ Transposase gene in the *B. thuringiensis* 407 genome

**Transposase gene**	**Copies**
IS*110* family transposase	12
IS*110*-like element ISBth13 family transposase	5
IS*21*-like element IS*232* family transposase	5
IS*3* family transposase	5
transposase	5
IS*4* family transposase	2
IS*4*-like element IS*231*A family transposase	2
IS*4*-like element IS*231*C family transposase	1
IS*607* family transposase	1
IS*66* family transposase	1

B/ Insertion sequence definition in the second-round analysis using *breseq*

IS no.	Position	Length	Tag	Gene name
IS1	1903509~1905263	1755	BTB_RS09780	IS*4* like element IS*231*A family transposase
IS2	2497927~2499656	1730	BTB_RS12605	IS*110* family transposase
IS3	2615786~2617440	1655	BTB_RS13165	IS*4* like element IS*231*A family transposase
